# Trigeminal Cardiac Reflex: New Thinking Model About the Definition Based on a Literature Review

**DOI:** 10.1097/MD.0000000000000484

**Published:** 2015-02-06

**Authors:** C. Meuwly, E. Golanov, T. Chowdhury, P. Erne, B. Schaller

**Affiliations:** From the University of Basel, Switzerland (CM); The Houston Methodist Research Institute, Houston, Texas, USA (EG); Department of Anesthesia and Perioperative Medicine. University of Manitoba, Winnipeg, Canada (TC); Cardiology, St Anna Clinic, Luzern, Switzerland and University of Basel, Switzerland (PE); Department of Research, University of Southampton, United Kingdom (BS); and Academic Editor, Medicine (BS).

## Abstract

Trigeminocardiac reflex (TCR) is a brainstem reflex that manifests as sudden onset of hemodynamic perturbation in blood pressure (MABP) and heart rate (HR), as apnea and as gastric hypermotility during stimulation of any branches of the trigeminal nerve. The molecular and clinical knowledge about the TCR is in a constant growth since 1999, what implies a current need of a review about its definition in this changing context.

Relevant literature was identified through searching in PubMed (MEDLINE) and Google scholar database for the terms TCR, oculocardiac reflex, diving reflex, vasovagale response.

The definition of the TCR varies in clinical as well as in research studies. The main difference applies the required change of MABP and sometimes also HR, which most varies between 10% and 20%. Due to this definition problem, we defined, related to actual literature, 2 major (plausibility, reversibility) and 2 minor criteria (repetition, prevention) for a more proper identification of the TCR in a clinical or research setting. Latest research implies that there is a need for a more extended classification with 2 additional subgroups, considering also the diving reflex and the brainstem reflex.

In this review, we highlighted criteria for proper definition and classification of the TCR in the light of increased knowledge and present a thinking model to overcome this complexity. Further we separately discussed the role of HR and MABP and their variation in this context. As another subtopic we gave attention to is the chronic TCR; a variant that is rarely seen in clinical medicine.

## INTRODUCTION

The trigeminal cardiac reflex (TCR) is a unique brainstem reflex that manifests as typical hemodynamic perturbations including sudden lowering of heart rate (HR), mean arterial blood pressure (MABP), cardiac arrhythmias, asystole, and other autonomic reactions such as apnea and gastric hypermotility. Some of these manifestations were initially, described as a peripheral airway reflex by Kratschmer.^1^ In the early 1990s, the oculocardiac reflex (OCR) that is presently accepted as one of the peripheral subtypes of the TCR, was described by Aschner D.^2^ The other pioneers of this field include Kumada and colleagues (who described the trigeminal-depression response), Shelly and Church (who coined the term TCR), and especially Schaller and colleagues (who first described the central component of the TCR in humans and have pioneered the definition/concept of the TCR in humans).^3–5^ Following the pioneering work of Schaller and colleagues in 1999, this unique reflex gained a lot of interest in the field of neurosurgery. In addition, Schaller and colleagues further explored the possibilities of different clinical variants of the TCR^6–22^ and presented the generally accepted classification of peripheral and central subtypes of the TCR.^23,24^

Contemporary research has revealed the possibility of additional subtypes of the TCR, recently seen during manipulation around the Gasserian ganglion.^25^ Moreover, the definition of the TCR is still a matter of clinical controversy and ongoing research. Therefore, this article aims to provide an update of knowledge and concepts in relation to the definition of the TCR presenting a new thinking model.

## TCR: A UNIQUE CLINICAL ENTITY

The TCR is a clinical phenomenon consisting of sudden onset of hemodynamic perturbations (acute changes in MABP, decreased HR, asystole), respiratory changes (apnea) and gastric changes (hypermotility) resulting from stimulation of any branch of the fifth cranial nerve along its course. This stimulation triggers the nerve to send neuronal signals via the Gasserian ganglion to the sensory nucleus of the trigeminal nerve. In the sensory nucleus, the signals are linked through a powerful excitatory and polysynaptic connection to the reticular formation. This connection seems to be endogenously modulated, differentially enhanced and depressed by 5-HT1A and 5-HT2A-receptors antagonists.^24–27^ This is considered as the afferent pathway of the reflex. Through short internuncial fibers to the reticular formation,^24^ the afferent pathway is connected to the efferent pathway of the reflex, which in significant part arises from parasympathetic neurons in the motor nucleus of the vagus nerve. The stimulation of the vagus nerve is responsible for bradycardia and hypotension as well as other manifestations including predominantly apnea and gastric hypermotility. Research on animals has also highlighted several other important nuclei related to TCR pathway, including the trigeminal nucleus caudalis, the parabrachial nucleus, the rostral ventrolateral medulla oblongata, the dorsal medullary reticular field and the paratrigeminal nucleus. Unusual manifestations of TCR such as hypertension or tachycardia point toward an activation of the sympathetic nervous system as well.^24,26,28,29^

## LITERATURE RESEARCH

**FIGURE 1:**
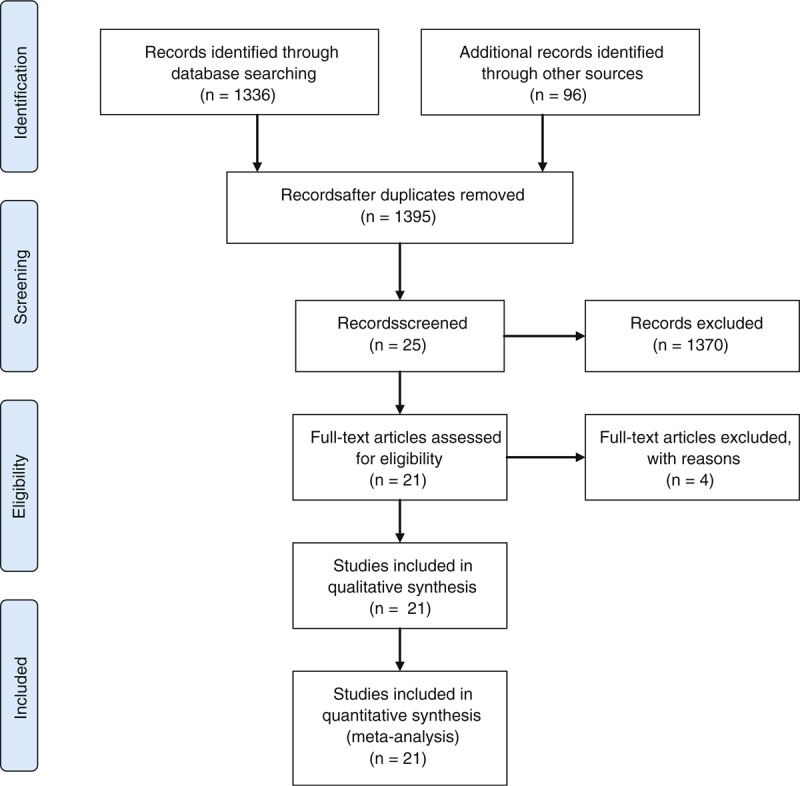
Flow Diagram of Different Phases of the Review according to PRISMA.

Relevant literature was identified through searching in PubMed (MEDLINE) and Google scholar database by searching for the terms “trigeminocardiac reflex,” “oculocardiac reflex,” “diving reflex,” “vasovagale response.” Relevant literature was chosen ad hoc according to definition and to classification of the TCR with the goal to create a comprehensive, widespread picture about the current meaning (see Figure 1).

TCR cases were defined as a drop in MABP and HR, both more than 20% to baseline levels (details of the definition see below) and had to fulfill at least 2 major criteria in plausibility and reversibility as described earlier by Schaller et al^5^ Data extraction was made by 2 independent reviewers and the prevalence was calculated by SSPS statistics.

## ETHICAL REVIEW

The present work does not need any ethical approval as it involves no interaction with human subjects or does not collect identifiable private information.

## DEFINITION OF TCR

**FIGURE 2:**
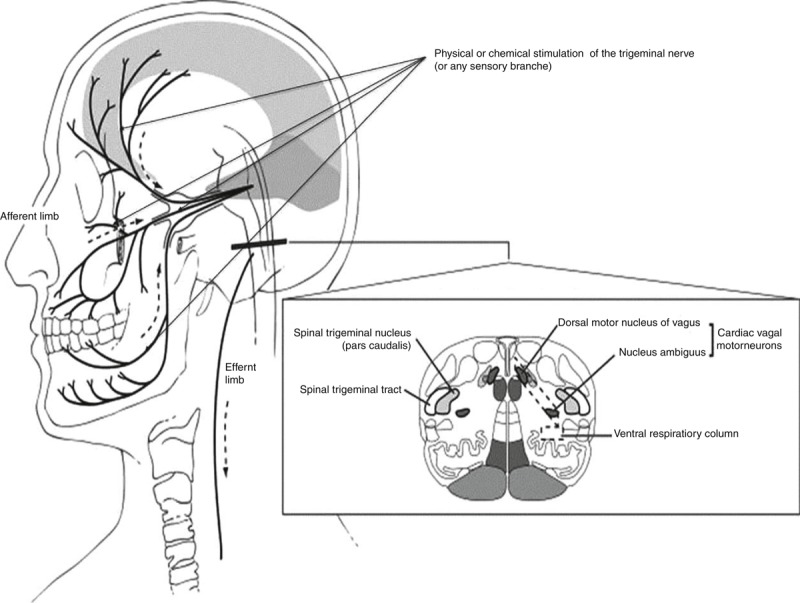
Pathway of the trigeminocardiac reflex.^23^

The TCR is commonly defined as suggested by Schaller and colleagues as a sudden drop in HR and MABP of more than 20% as compared with baseline values (see Figure 2),^5^ evoked by a physical (mechanical, electrical) or chemical manipulation of any of the branches of the trigeminal nerve.^30,31^ Any other sudden autonomic response, with or without hemodynamic changes, as a reaction to the stimulation of the trigeminal nerve on any point of its course are considered as a trigemino*vagal* reflex (TVR).^24,26^ It should be noted that the TCR was originally coined to describe concrete autonomic changes upon stimulation of the trigeminal nerve.^32^ None of the clinical assessments of the TCR, to the best of our knowledge, have hitherto concerned this complex picture.^32^

There is therefore an ongoing discussion about the size of hemodynamic changes to define the event as a TCR, and some authors consider the reflex as a change of 10% compared to the baseline values.^33^ The arbitrary definition of 20% decrease based on pre-procedural levels leading to the TCR risks underestimating the incidence of the TCR; however, it decreases the risks associated with trivial baseline changes, often associated with anesthesia and the patient's positioning, and more effectively excludes cardiovascular disturbances not unusually seen during surgery not related to the reflex.^32^ At what point and at what state HR and MABP is measured, was too diverse to generate any generalization from the currently included literature.

According to Bohluli et al^34^ who systematically studied the occurrence of the TCR in maxillofacial procedures, a mean decrease of 6.5% and 9.7% compared with baseline was recorded for Le-Fort I osteotomy, respectively in mean HR and MABP. Also in bilateral sagittal splitting ramus osteotomy, the locally blocked side was associated with mean decreases of 4.6% of HR and 6.8% of MABP during splitting and manipulation, compared with baseline while these values were 17.6% and 21.5% in the control ramus.^34^ Therefore, it seems that the artificial 20% cut-off only results in inclusion of the actual cases of TCR episodes and prevents false positive case inclusion.^32^ Since the magnitude of the reflex, seems to be evoked by surgical maneuvers, the rate may be much higher than what has been described in the literature.^32^ However, the suggested change of 20% is used most often in current publications and seems to be reasonable from a statistical and research point of view. This definition remains arbitrary, of course, but it helps to exclude false-positive cases. Importantly, based on the present classification of the TCR into different subtypes, the definition should be explored and adapted to each subtype of the TCR. The traditional classification by Schaller et al (a change of HR and MABP of 20%) still seems reasonable for the central reflex. But, considering that the peripheral reflex does manifest as wide array of cardiovascular as well as autonomic symptoms, and does not necessarily involve a decrease in MABP,^24,26,35^ the definition should be modified accordingly. On the other hand, bradycardia is still an important component of the peripheral reflex, and a change in HR of 20% seems reasonable. Overall, the most important aspect of the definition remains that the reflex incitation should be a reaction to a physical (mechanical, electrical) or chemical stimulus applied to any branch of the trigeminal nerve after excluding the reaction to pain. In most of the reported literature, the hemodynamic changes are reversible after abolition of the stimulus that evoked the TCR.

## CAUSE–EFFECT-RELATIONSHIP

It is important to stress here that not every bradycardia is a TCR. Besides formal definition as described above, there is a need to define a clear “cause-/-effect” relationship.^5^ If clear unequivocal definitions are used in every published case (see Table 1), comparison of the works related to TCR would be more feasible and consequently systematic reviews would help to create new knowledge.

**TABLE 1:**
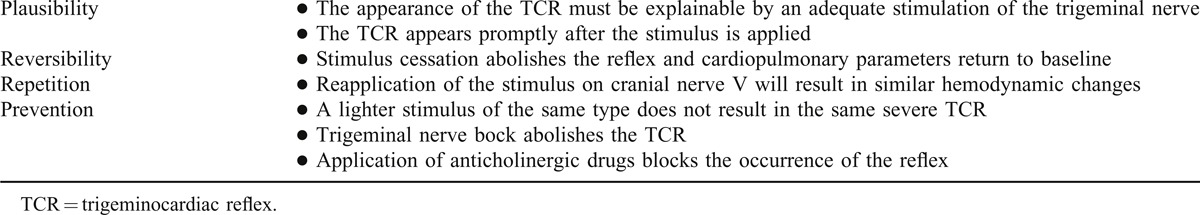
Evidence of TCR by Cause–Effect Relationship

The TCR is elicited by the maneuvers around any of the branches of cranial nerve V or upon stimulation of Gasserian ganglion or trigeminal brainstem centres.^32^ A direct cause-and-effect assessment of the trigger point of the reflex, however, is ethically not possible in humans and requires exploration in animal models. Such a generally accepted and clinically driven model that is properly and sufficiently studied presently is lacking.^32^ The criteria necessary [described by Blanc as well as Schaller] to establish such a cause–effect relationship in a clinical setting should include the consistency and strength of the association, the presence of a type of stimulus–incidence relationship, and biological plausibility.^5,37^ It is important to understand that according to these criteria an elimination of the inducing stimulus has to reverse the reflex-induced changes whereas repetition of the stimulus should provoke the reflex each time.^5,36^ In daily practice, such a cause–effect association can be anticipated by the effectiveness of measures used to prevent the reflex.^5^ In this context, we have to take into consideration that repetitive stimulus might modify the reflex by supraspinal influences such as habituation, peripheral adaption or fatigue of receptors. The preventative strategies include avoidance of the stimulation of branches of the trigeminal nerve, blocking of the nerve by local anesthetics (not an absolute method), and prior administration of anticholinergic drugs (not an absolute method).^5^ Although there are few reported cases^37,38^ of the patients who showed the TCR-related effect even after cessation of the stimulus and needed to be treated by cardiac massage, the reversibility should be included in the TCR definition as a necessary criterion.^5^ The present review has shed light on a new and important point: The time lag between the stimulus and the response in form of the TCR. From the current review we have therefore defined that a time lag up to 5 seconds would be appropriate for a positive cause–effect relation (98–99% confidence interval).

These 5 principal points would help in clinical practice to assess whether the observed hemodynamic changes are related to a TCR-phenomenon. In daily clinical practice, not all the criteria must/can be always required to confirm a TCR. But the more of these criteria are present the more confirmed is a TCR. As described above, an assessment of the reversibility of the TCR can ethically not be tested, but is observed under some circumstances.^39^ The prevention of the TCR does not represent an absolute criterion because several cases have been described in the literature where neither blockade of the trigeminal nerve nor the application of anticholinergic drugs/local anesthetics ceased the occurrence of the reflex.^40,41^ Moreover, such an approach makes no sense from a pathophysiological point of view as the reflex originates central to the site of the blockade. We can therefore define 2 major criteria as plausibility and reversibility as well as 2 minor criteria as repetition and prevention. Aside from the percentage drop of MABP and HR (as described above), the 2 major criteria should be observed for a sound definition of TCR. The 2 minor may not be observed in a TCR-event, dependent on the different circumstances in which the TCR occurs.

This cascade of evidence is important for further publications in this field; especially to create a pool of relevant scientific data that can be used to exclude substantial biases on TCR related research/systemic reviews.^42^

## TRADITIONAL CLASSIFICATION PERIPHERAL-GANGLION GASSERI-CENTRAL

The traditional classification of the TCR is based on the location of the trigger point (see Table 2). A central (proximal) TCR is triggered by the stimulation upon the intracranial part of the trigeminal nerve, thus upon the section which is located after the Gasserian ganglion. A peripheral (distal) TCR is therefore triggered by stimulation upon the extra-cranial course of the trigeminal nerve, distal to the Gasserian ganglion. The peripheral TCR is further subdivided based on the branch of the affected trigeminal nerve into the oculo-cardiac reflex (V1) and the maxilla-mandibulo-cardiac reflex (V2–V3). A TCR, triggered at the Gasserian ganglion has, according to the latest studies,^23,25^ its own entity and is classified as a separate subtype.^25^ In all the subtypes, apnea and gastric hypermobility are common manifestations. The clinical presentation of the peripheral and the central subtype is almost identical. Both subtypes present a slowdown in HR. In the central TCR, a decrease in MABP is always seen and is considered as necessary while in the peripheral TCR, a change in MABP is not always observed. The oculo-cardiac reflex often observed during traction on extra-ocular muscles is usually associated with cardio-depressive effects whereas the maxilla-mandibular subtype of the peripheral TCR shows predominantly *vagal responses*. In both subtypes, a sympathetic co-activation can occur after stimulation of the infra-orbital nerve.^25,43^

**TABLE 2:**
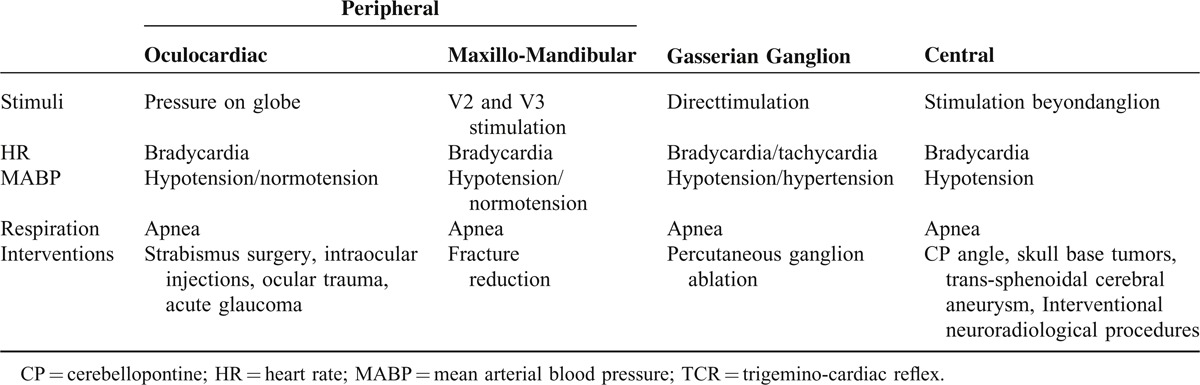
The Traditional Classification of the TCR

Further, a TCR of the “Gasserian ganglion” subtype is defined as a reaction to direct stimulation around the ganglion, clinically presented as a change in HR and/or MABP in decreasing or increasing both parameters. The Gasserian ganglion is located in Meckel's cave and its neurons innervate mechanoreceptors, thermoreceptors and nociceptors; it has therefore mostly a sensory function (information from V1, V2, V3) but it has also motoric components (V3 only). On the medial side, the ganglion passes sympathetic fibers from the carotid plexus. Considering this complex composition of parasympathetic and sympathetic fibers, it seems understandable why the TCR presents in different symptoms.^44–46^ With these physiologic mechanisms in mind, we can suppose that the clinical presentation of the TCR can result in activation of the sympathetic as well as the parasympathetic nerve system. Thus it seems as the peripheral TCR has more over-shooting sympathetic components than the central TCR. In this context, the often seen normotension in peripheral TCR could be explained by peripheral vasoconstriction due to sympathetic stimulation similar to the diving reflex (DR).

A TCR of the Gasserian ganglion type seems not to have a characteristic presentation presumely because the balance between parasympathetic and sympathetic influences differs from 1 patient to another.^25^ The differences between central and peripheral TCR may arise due to the integrative properties of the ganglion neurones receiving multimodal information and innervating numerous brainstem sites, resulting in different manifestations.^26,35^

The published cases of TCR during manipulation at the Gasserian ganglion described various changes of hemodynamic parameters, such as bradycardia and *hypo*tension upon entry of the needle into the foramen ovale,^47^ as well as tachycardia and *hyper*tension after compression of the ganglion but abrupt bradycardia after puncture of the foramen ovale.^48^ The origin of this reaction is not yet clear and could be because of stimulation of fibers of passage or of a general trigeminal (nociceptive) sympathetic defence response.

In addition, the findings of Chen and colleagues have shown that bradycardia with a simultaneous increase of MABP after percutaneous microballoon compression of the trigeminal ganglion can be prevented with anticholinergic drugs (eg, atropine) or with labetolol (a mixed α/β adrenoceptor antagonist).^50^ The fact that severe bradycardia and hypertension were prevented by labetolol underlines a likely imbalance between sympathetic and parasympathetic influences during a TCR episode. As a non-selective beta-adrenergic receptor blocker and selective alpha1 receptor blocker, labetolol decreases hypertension by blocking adrenergic stimulation of beta-receptors within the myocardium and alpha1-receptors within vascular smooth muscles.^49^ The fact that labetolol had a preventative effect on the TCR implies the co-activation of the sympathetic nerve system. This co-activation has also been shown for the peripheral TCR by Nalivaiko et al^50^ where the nasopharyngeal reflex was stimulated by inhalation of formaldehyde vapor, resulting in abrupt bradycardia. After treatment with muscarinic cholinergic blockade (with methylscopolamine), a small tachycardia response was unmasked in 5/7 animals. They concluded that there were “ […] increased vagal effects in the sino-atrial node, and increased sympathetic effects in the ventricular myocardium."^50^

## EXTENDED CLASSIFICATION: TOWARDS THE COMPREHENSIVE UNDERSTANDING OF THE TCR

The latest research has shown a similar reflex arc in the TCR and the DR (see Table 3).^51,52^ The DR manifests as breath-holding, slowing down of the HR, decreased cardiac output, peripheral vasoconstriction and increased MABP.^53,54^ Both the TCR and the DR, are phylogenetically old oxygen preserving reflexes and they are most important and most often seen in newborns and infants where it causes a decrease in HR from 5% to 51% by a single facial submersion.^27,51–56^ The DR is triggered by stimulation of the forehead or nasal mucosa with cold water or cold air-flow, which are innervated by the V1 subdivision of the trigeminal nerve. The shared pathways suggest that the DR is another, very peripheral, sub-classification of the TCR.^25,55^ The difference between the peripheral TCR and the DR is mainly the reaction on the MABP. Regarding the changes in MABP, the peripheral TCR causes most often normo-/or hypotension and bradycardia, the DR provokes peripheral vasoconstriction resulting in hypertension accompanied by bradycardia.^27^ The physiological difference between the peripheral TCR and the DR is the balance between stimulation of the (para-) sympathetic nerve system. While the peripheral variant of the TCR results in normo- or *hypo*tension, the DR clinically presents as *hyper*tension. Thus, we can assume that the DR has a stronger activation of the vascular sympathetic nerves than the peripheral TCR.

**TABLE 3:**
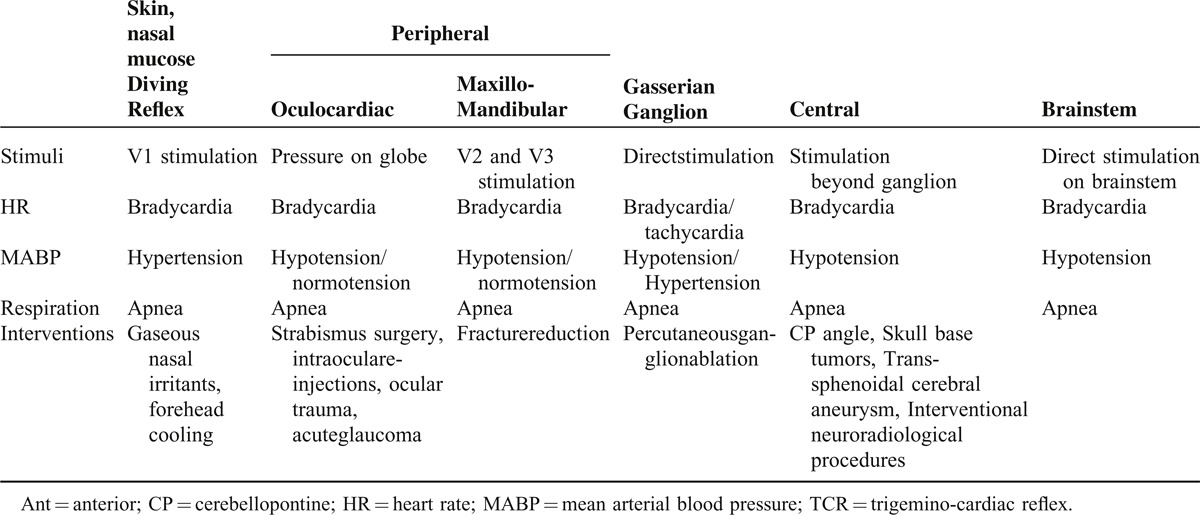
The Extended Classification of the TCR

To complete this account, a TCR triggered at the central part of the trigeminal nerve has a strong representation of parasympathetic influences that presents clinically as a decrease in MABP and HR.

## THE ROLE OF HEART RATE IN THIS CONTEXT

HR is regulated by the sympathetic and the parasympathetic input to the sinoatrial node. The last part of the reflex arc of the TCR is formed by cardio-inhibitory efferent fibers, which connect the motor nucleus of the vagus nerve to the myocardium.^57^ The cardioinhibitory fibers are mainly distributed to the sinoatrial and the atrioventricular nodes and not to the ventricles. Therefore, the main effect of the vagal nerve is chronotropic. All variants of the TCR cause bradycardia except the Gasserian ganglion type, where brady-/or tachycardia can be observed.^25^ TCR often results in a major form of a parasympathetic onset that clinically presents as severe bradycardia or asystole. During an episode of TCR, the parasympathetic nerve activation prevails over the cardiac sympathetic stimulation; which suppresses the tachycardic response to the decrease of MABP resulting from a peripheral vasodilatation (most often seen in central TCR cases).

**FIGURE 3:**
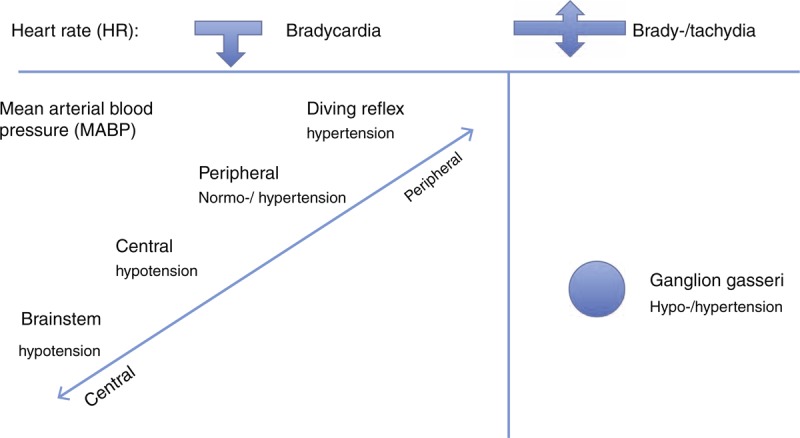
Thinking model about the TCR. HR = heart rate; MABP = mean arterial pressure; TCR = trigeminocardiac reflex.

Although the cardioinhibitory fibers of the vagus nerve are activated in every TCR, the effect on MABP varies depending on where the exact trigger point of the TCR is located. The MABP can even increase (DR) even though the cardiodepressive vagus fibers are activated because of the simultaneous activation of the sympathetic nerve system and arterial baroreceptors, which provoke vasoconstriction of small blood vessels (see Figure 3). These differences may arise from the different nature of TCR triggered by direct nerve stimulation and DR triggered by stimulation of specific skin or mucosal receptors. In the first case the response is triggered by stimulation of nonspecific nerve fibers, while DR triggers a complex physiological response to activation of specific receptors.

The recent work of Chowdhury et al^25^ has further shed light on these differences and has especially pointed out the different role of the Ganglion Gasseri.

## THE ROLE OF BLOOD PRESSURE IN THIS CONTEXT

Arterial blood pressure is one the most highly regulated hemodynamic variables in humans. It is clear that, in addition to arterial baroreceptors^58–61^ located in the central arteries and the aorta, many additional autonomic reflex loops can serve to regulate blood pressure. Evidence in animals suggests that afferent nerve endings in peripheral veins may sense regional blood volume changes and, in turn, contribute to the blood pressure regulation via a reflex response in animals.^55,62–64^ Regarding the effect of the TCR on MABP, it is important to differentiate the variants of the TCR. As observed in daily practice, a TCR of the peripheral type does not have the same strong inhibitory effect on the MABP as a TCR of the central type (see above). Smaller hypotensive potential (of the peripheral types of TCR) probably results from activation of different co-stimulation of sympathetic and parasympathetic reflexes. Further studies are needed to gain further insights into these differences.

## CHRONIC VERSUS ACUTE TCR

The most often clinically observed form of the TCR is correlated with an acute onset and a very short duration. Nearly all our knowledge about this brainstem reflex is based on the acute form.

A chronic form of the TCR, mostly observed as the OCR, has also been reported.^65–68^ Spiriev et al^65^ described 2 cases with manifested TCR-episodes days after the successful microsurgical clipping of a symptomatic intracranial aneurysm (patient 1 on days 10 and 12 and patient 2 on days 2 and 5 after surgery). Another case of a chronic onset of the TCR was published by Chowdhury et al^66^ in which a patient with an orbital floor fracture presented symptoms of the TCR even after 1 month. These symptoms improved dramatically after surgery on the orbital floor, which ceased the stimulation of the cranial nerve V1 and therefore the resulting OCR. The probable longest time to onset a TCR reported in literature was presented by Yang et al^67^ They described a case of chronic OCR, which occurred with a delay of 40 years and was triggered by an intraorbital foreign body. These few case reports demonstrate that chronic variants of the TCR might be substantially underdiagnosed. Probably some postoperative complications in long-term follow-up might be due to the TCR. Although research on the chronic TCR is relatively new it has already shown that a permanent stimulation of the trigeminal nerve can lead to substantial deficits.^69^ Such cases highlight the importance of further studies about the outcome and long-time effects of the TCR (acute and chronic).

## DISCUSSION

In the present era, the role of TCR in different neurosurgical conditions, its mechanisms, risk factors, prevention and management are being highlighted. However, the need of standard definition of a TCR episode cannot be ignored in view of promoting the consistency of clinical studies/research. The common criteria of at least a 20% fall (or clear increase) in MABP as well as HR is certainly clinically driven but seems reasonable as this represents major TCR events and may eliminate anesthesia and other physiological changes. Also our present thinking models ignores the others autonomic responses such as apnea or gastric motility. On the other hand, the subtle TCR events may be missed using this relative strict definition. Therefore, the actual incidence of TCR episodes may be even higher. In addition, narrow range (10% or less change in MABP/HR) may be a potential early warning sign that can prevent fatal TCR episodes and related injuries. Unfortunately, as the narrow-range definition cannot precisely delineate TCR episodes from other factors, it seems inappropriate to adopt the latter definition. The numerous cases seen since 1999,^5^ however, let us conclude that fall in HR is much a more precise sign for TCR than it is MABP; because of the variability of coincident sympathetic influences of the latter.

Literature reviews have always its limitations as one can only study what is already published. In a topic like the TCR in which predominantly cases and case series are published, there is additionally the limitation of a positive group selection what often hinders the generalization of the results. We have overcome this limitation that results in a substantial complexity by our thinking model. The second principal limitation point is that we several important regions, like the Meckel's cave, in which no information about the TCR exists so far. Last, the currently neurosurgical driven TCR research with predominant anaesthetized patients excludes the research on other autonomic responses of the TCR such as apnea or gastric motility. Especially the aspect of apnea would first open our understanding of the TCR as a rather pan-autonomic response instead as only referring to system effects as actually done.^32^

In recent times, literature on the TCR has provided substantial evidence on the different subtypes of TCR. Out of these, the 3 main important subtypes include peripheral, central and Gasserian Ganglion- type TCR. As hemodynamic changes have different presentations in different subtypes, it is not feasible to use a definition based on the smaller changes of HR/MABP. Therefore, our article presents the unique cause–effect relationship based criteria for TCR episode and highlights 4 major domains including plausibility, reversibility, repetition, and prevention. Further to this, we have divided these 4 major criteria to 2 major (plausibility and reversibility) and 2 minor criteria (repetition and prevention). For defining TCR, there should be at least 2 major criteria. Minor criteria may or may not be met in all cases. This dynamic criterion would certainly provide relevant research and studies in the field of TCR in the near future. Even the TCR is currently restricted to neurosurgical procedures; better understanding of the underlying factors will certainly open the doors neurological diseases affecting the autonomic nervous system.

## CONCLUSION

In summary, objective definitions of TCR are still not absolute, and have their own drawbacks. Moreover, the different sub types of TCR also manifest differently and these definitions cannot be applied uniformly. Therefore, our article for the first time presents a thinking model of dynamic criteria to define the complex TCR episodes irrespective of the subtypes that will serve to provide a platform to cite the TCR events appropriately, and form the future pathways for more relevant research/studies on TCR based on these criteria.
